# Factors Associated with Long COVID in the Pediatric Population: A Retrospective Case–Control Study

**DOI:** 10.3390/clinpract16060105

**Published:** 2026-05-31

**Authors:** Ioana Maria Otilia Lică, Iulia Florentina Țincu, Anca Cristina Drăgănescu, Doina Anca Pleșca

**Affiliations:** 1 Pediatric Department, University of Medicine and Pharmacy “Carol Davila”, 020021 Bucharest, Romania; ioana-maria-otilia.lica@drd.umfcd.ro (I.M.O.L.); doina.plesca@umfcd.ro (D.A.P.); 2 “Dr. Victor Gomoiu” Children’s Clinical Hospital, 022102 Bucharest, Romania; 3 National Institute of Infectious Diseases “Prof. Dr. Matei Balș”, 021105 Bucharest, Romania

**Keywords:** long COVID, children, SARS-CoV-2 infection, laboratory markers, risk stratification, pediatric cohort

## Abstract

**Background:** Long COVID in children is increasingly recognized, yet its clinical predictors and objective biological correlates remain insufficiently characterized. **Objectives:** The objective was to compare clinical, demographic, and laboratory characteristics between children with and without long COVID and to identify associated variables. **Methods:** We conducted a retrospective observational case–control study at the “Dr. Victor Gomoiu” Children’s Clinical Hospital, including pediatric patients with confirmed SARS-CoV-2 infection. Cases were defined as children with symptoms persisting ≥12 weeks after acute infection, while controls had no persistent symptoms at ≥12 weeks. **Results:** Eighty-nine children with long COVID and 88 matched controls were included. Children with long COVID were significantly older (1.79 ± 0.90 vs. 1.14 ± 0.80 years, *p* < 0.001) and more frequently from urban areas (86.5% vs. 69.3%, *p* = 0.0099). Lymphocyte, monocyte, and basophil counts were significantly lower in the Long COVID group, while D-dimer, ferritin, serum iron, urea, and creatinine levels were significantly higher. A multivariate predictive model demonstrated excellent discrimination (AUC = 0.94), with optimal sensitivity (84.3%) and specificity (89.8%) at a probability threshold of 0.48. **Conclusions:** Long COVID in children was associated with identifiable clinicobiological features. An exploratory composite model showed good discrimination but requires external validation.

## 1. Introduction

Although children generally experience milder acute SARS-CoV-2 infection, a subset develops persistent symptoms lasting beyond the acute phase, referred to as post-COVID-19 syndrome or long COVID. According to the World Health Organization, symptoms associated with long COVID condition in children may substantially interfere with daily functioning, including eating patterns, physical activity, behavior, school performance, social interactions, and developmental progress [1]. These manifestations may either emerge after apparent recovery from the acute infection or persist from the initial illness and can show a relapsing or fluctuating course over time [1]. The heterogeneity of clinical presentation and the absence of validated pediatric risk stratification tools complicate follow-up strategies. Identifying factors associated with long COVID may improve early recognition and targeted monitoring in pediatric populations. In the available literature, the reported prevalence of long COVID syndrome (LCS) is consistently higher in adults than in children. In a recent retrospective longitudinal study including 436 Italian adult inpatients and outpatients with a prior diagnosis of COVID-19, long COVID was identified in 71.8% of participants [2]. Moreover, a comprehensive meta-analysis encompassing 1,680,003 hospitalized and non-hospitalized COVID-19 patients worldwide estimated a global long COVID prevalence of 42%; however, the analyzed populations were predominantly composed of adults [3]. A nationwide cross-sectional study was conducted, including pediatric patients with PCR-confirmed SARS-CoV-2 infection and a control group drawn from Danish national population registers. Eligible participants were children aged 0–14 years who tested positive between 1 January 2020 and 12 July 2021 [4]. Data were collected through proxy-reported questionnaires completed by mothers. Health-related quality of life and somatic symptom burden were assessed using validated instruments, namely the Pediatric Quality of Life Inventory (PedsQL) and the Children’s Somatic Symptoms Inventory-24 (CSSI-24); in addition, supplementary items were included to capture the presence of the 23 most commonly reported long COVID symptoms. The conclusions of the study were that children with previous SARS-CoV-2 infection showed a higher prevalence of persistent symptoms than controls, supporting the recognition of pediatric long COVID and the need for multidisciplinary follow-up care [4]. This is similar to previous research in pediatric chronic conditions, such as ADHD, that identified multiple factors that negatively influence quality of life, highlighting the multifactorial nature of long-term functional impairment [5].

The relevance of standardized clinical algorithms and structured documentation in pediatric practice has been emphasized in European multinational research [6], highlighting the impact of non-uniform protocols on case identification and management consistency. Analogously, the heterogeneity currently observed in pediatric long COVID definitions, diagnostic pathways, and follow-up strategies supports the need for validated predictive models and harmonized risk-stratification frameworks to improve reproducibility, comparability across centers, and clinical decision-making.

Early nutritional exposures have been shown to modulate immune maturation and long-term health outcomes in children, potentially influencing susceptibility to infectious and post-infectious conditions [7]. Early-life biological and nutritional factors are known to influence immune development, metabolic regulation, and disease susceptibility in pediatric populations. Previous studies in Romanian infant cohorts have demonstrated that feeding practices and protein intake during the first year of life are associated with growth patterns and circulating insulin-like growth factor I (IGF-I) levels, underscoring the long-term impact of early nutritional exposures on biological trajectories [8,9]. Emerging evidence suggests that host-related biological factors, including immune and metabolic programming, may also modulate susceptibility to SARS-CoV-2 infection and the risk of post-acute sequelae, including long COVID, in pediatric populations [10].

A large multicenter prospective cohort study conducted in 14 tertiary pediatric emergency departments in Canada evaluated the prevalence of long COVID among children tested for SARS-CoV-2 between August 2020 and February 2022, with follow-up assessments at 6 and 12 months [11]. The authors concluded that although persistent symptoms were more common after SARS-CoV-2 infection, the overall prevalence of long COVID in this pediatric population was low and was not associated with a measurable long-term reduction in quality of life. Long-term consequences of COVID-19 have been mainly described in adults from high-income countries, whereas evidence in children remains limited. A recent systematic review including over 825,000 pediatric participants reported highly variable prevalence estimates of long COVID condition, largely based on heterogeneous definitions and symptom persistence. Fatigue, headache, and respiratory symptoms were most frequently reported [12].

This study aimed to compare clinical, demographic, and paraclinical features of pediatric patients with and without long COVID and to identify associated risk factors. Secondary aims included describing paraclinical investigation patterns and developing an exploratory predictive model for pediatric long COVID.

## 2. Materials and Methods

### Study Design and Population

This retrospective observational case–control study was conducted at the “Dr. Victor Gomoiu” Children’s Clinical Hospital, Bucharest, Romania. Pediatric patients, aged between 1 month and 18 years old, with confirmed SARS-CoV-2 infection (antigen or RT-PCR) between January 2021 and December 2022 were eligible. Groups were divided as follows: Group 1 (long COVID) were children presenting with persistent symptoms lasting ≥12 weeks after acute COVID-19 (89 individuals) and Group 2 (Controls) were children with confirmed SARS-CoV-2 infection who did not develop persistent symptoms at ≥12 weeks, matched to cases in a 1:1 ratio based on: age (±1 year in young children, ±2 years in adolescents), sex, year of infection (88 individuals). Inclusion criteria included (1) confirmed COVID-19 infection through polymerase chain reaction (PCR) or rapid antigen testing; (2) documented symptom follow-up at ≥12 weeks; (3) signed informed consent for participation provided by the legal guardian. Exclusion criteria comprised (1) patients with severe chronic diseases potentially confounding symptom attribution; (2) refusal of the parents to participate; (3) patients with incomplete follow-up data were excluded. The collected data were analyzed using IBM SPSS Statistics 30.0.0.0.

*Variables*. Data collected in this study included: Category (I): age at diagnosis (years), gender (male/female), residence (urban/rural), co-morbidities (yes/no), SARS-CoV-2 vaccination status (yes/no), COVID-19 severity classified according to WHO guidelines, meaning distinguishing asymptomatic, mild, moderate, severe, and critical cases [13]. Category (II): haematological, inflammatory (C-reactive protein, fibrinogen, erythrocyte sedimentation rate (ESR), ferritin), metabolic (liver enzymes, creatinine, urea, iron status), and coagulation parameters (D-dimers); Category (III): data collected regarding lung ultrasound, abdominal ultrasound, echocardiography, chest X-ray, computer tomography or magnetic resonance imaging.

*Statistical Analysis.* Continuous variables were expressed as mean ± SD and compared using Student’s *t*-test. Categorical variables were compared using χ^2^ or Fisher’s exact test. Multivariate logistic regression was used to construct an exploratory predictive model. Model performance was assessed using ROC curve analysis. Statistical significance was set at *p* < 0.05. For the exploratory predictive score for long COVID, the authors used multivariate logistic regression, with long COVID status as the dependent variable. Independent variables were selected based on clinical relevance, univariate analysis results, and pathophysiological plausibility. The model included clinical-demographic variables (age, residence, comorbidities, acute COVID-19 severity) and selected biological markers (lymphocytes, monocytes, D-dimer, ferritin, creatinine). Regression coefficients (β), odds ratios (OR), 95% confidence intervals, and *p* values were calculated for each predictor. Model performance was assessed using ROC curve analysis, with the area under the curve (AUC) used as a measure of discriminative ability.

*Ethical Considerations.* The study was conducted in accordance with the Declaration of Helsinki and approved by the Ethics Committee of the “Dr. Victor Gomoiu” Children’s Clinical Hospital (approval no. 6648/03.04.2024).

## 3. Results

### 3.1. General Characteristics

The mean age of patients in the long COVID group was significantly higher compared to controls (1.79 ± 0.90 years vs. 1.14 ± 0.80 years, *p* = 0.001), suggesting a possible association between increasing age and the risk of developing persistent long COVID symptoms (Table 1). Sex distribution did not differ significantly between the two groups (*p* = 0.41), indicating no apparent influence of sex on the occurrence of long COVID in the studied pediatric population (Table 1). In contrast, the place of residence differed significantly between groups, with a higher proportion of children from urban areas in the long COVID group compared to controls (86.5% vs. 69.3%, *p* = 0.0099). Comorbidities were more frequently observed among children with long COVID (33.7% vs. 12.5%); however, this difference did not reach statistical significance (*p* = 0.071), suggesting a potential trend that may become relevant in larger cohorts (Table 1). No significant differences were observed between the two groups regarding COVID-19 vaccination status (*p* = 1.00), likely reflecting the very low uptake of anti-COVID-19 vaccination within the study population (Table 1). In contrast, adherence to the national routine childhood immunization schedule was high in both groups, with the majority of children being fully vaccinated according to age (98.9% in the long COVID group vs. 92.0% in the control group), without statistically significant differences (*p* = 0.071).

**Figure 1:**
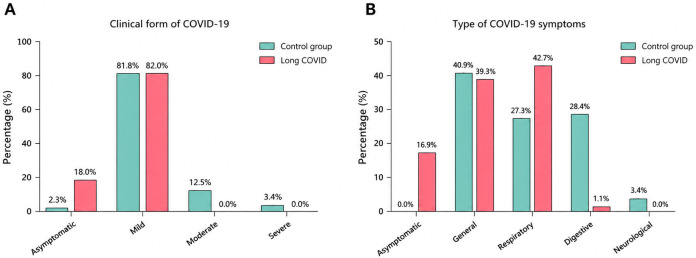
Clinical form and symptom profile of COVID-19. (**A**) Distribution of clinical forms of COVID-19 in the Long COVID and control groups. (**B**) Distribution of COVID-19 symptom types in the Long COVID and control groups.

The distribution of acute COVID-19 severity differed significantly between the two groups (*p* < 0.001). Children who later developed long COVID more frequently experienced asymptomatic or mild acute infections (18.0% asymptomatic and 82.0% mild forms), whereas moderate and severe forms were more commonly observed in the control group (12.5% moderate and 3.4% severe cases). Moreover, the type of acute COVID-19 symptoms differed significantly between groups (*p* < 0.001). In the long COVID group, respiratory (42.7%) and general symptoms (39.3%) predominated, whereas digestive (28.4%) and neurological symptoms (3.4%) were more frequently reported in the control group. Neurological manifestations were rare or absent among children with long COVID (Figure 1).

### 3.2. Laboratory Findings

Comparative analysis of biological parameters revealed statistically significant differences in several hematological, inflammatory, and metabolic markers between children with long COVID and controls. Children in the long COVID group exhibited significantly lower lymphocyte counts compared to controls (3.85 ± 1.47 vs. 4.51 ± 1.88, *p* = 0.010), as well as significantly lower monocyte and basophil counts (*p* < 0.0001), suggesting persistent alterations in cellular immune responses following SARS-CoV-2 infection (Table 2). Regarding inflammatory and coagulation parameters, D-dimer levels were significantly higher in the long COVID group (51.07 ± 123.61 vs. 10.39 ± 46.24, *p* = 0.004), indicating a possible persistent subclinical proinflammatory or procoagulant state. Serum ferritin and iron levels were also significantly higher in children with long COVID (*p* < 0.001 and *p* = 0.001, respectively), supporting the hypothesis of low-grade chronic inflammation or dysregulation of iron metabolism (Table 2). Hemoglobin levels were significantly higher in the long COVID group compared to controls (12.59 ± 0.70 vs. 11.10 ± 1.18, *p* < 0.0001), a finding that may be influenced by age distribution or inflammatory status. No statistically significant differences were observed between groups for total leukocyte count, neutrophils, eosinophils, platelets, CRP, ESR, fibrinogen, or liver enzymes (ALT, AST), suggesting the absence of ongoing systemic acute inflammation at the time of evaluation. Renal parameters showed significantly higher urea and creatinine levels in the long COVID group (*p* = 0.049 and *p* = 0.001, respectively); however, values remained within age-adjusted physiological ranges, warranting cautious interpretation and clinical correlation (Table 2).

### 3.3. Paraclinical Imaging Investigations

**Figure 2:**
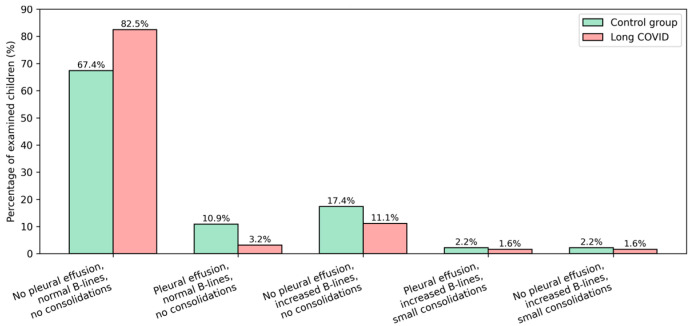
Distribution of lung ultrasound findings in children with long COVID and controls.

Lung ultrasound was performed significantly more frequently in children with long COVID compared to controls (70.8% vs. 52.3%, *p* = 0.017), reflecting the need for more extensive respiratory imaging in the context of persistent long COVID symptoms. Chest radiography was performed exclusively in the long COVID group (10.1% vs. 0%, *p* = 0.003), suggesting a higher clinical suspicion of residual pulmonary involvement in these patients. In contrast, abdominal ultrasound was significantly more frequently performed in the control group (83.0% vs. 20.2%, *p* < 0.0001), likely related to acute or non-COVID-related gastrointestinal conditions requiring imaging evaluation. No statistically significant differences were observed between groups regarding the use of echocardiography (*p* = 0.22) or advanced imaging modalities such as CT or MRI (*p* = 0.50). The distribution of lung ultrasound scores differed between children with long COVID and controls but did not reach statistical significance (*p* = 0.064). The most frequent category in the long COVID group was a normal ultrasound appearance, characterized by the absence of pleural effusion and a normal number of B-lines. In the control group, a higher proportion of examinations were not performed. Ultrasound findings suggestive of pulmonary abnormalities (pleural effusion, increased B-lines, and/or consolidations) were rare in both groups and showed a comparable distribution (Figure 2). After recoding lung ultrasound scores into binary categories (normal vs. abnormal), no statistically significant differences were observed between groups (*p* = 0.50). The majority of children in both groups had normal lung ultrasound findings or did not require ultrasound evaluation.

### 3.4. Predictive Model for Long COVID

The optimal probability threshold for classification, determined using the Youden index, was *p* (long COVID) ≥ 0.48. At this cut-off, the model demonstrated a sensitivity of 84.3% and a specificity of 89.8%, with a positive predictive value of 89.3% and a negative predictive value of 84.9%. Table 3 presents the variables included in the multivariable logistic regression model used to construct the long COVID prediction score. Biological variables showed a major contribution to the predictive performance of the model, alongside clinical factors such as comorbidities. The regression coefficients and odds ratios reflect the direction and magnitude of the associations between each variable and the probability of long COVID.

**Figure 3:**
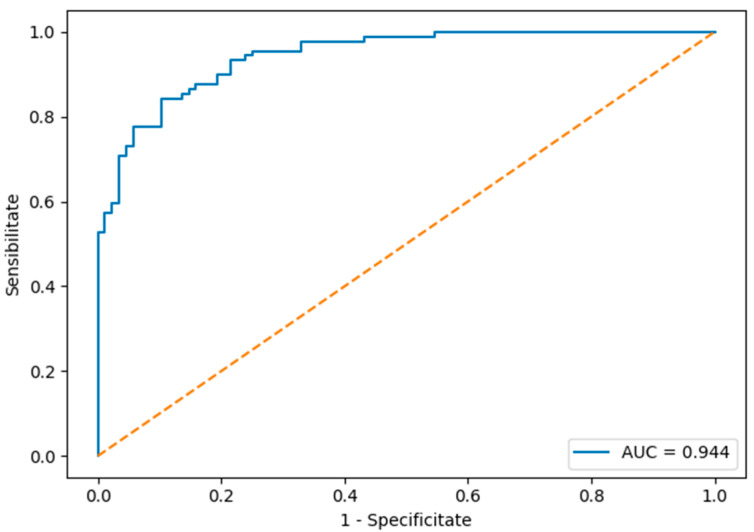
ROC curve for the long COVID prediction model.

The multivariate logistic regression model demonstrated excellent discriminative performance, with an AUC of 0.94 (Figure 3), indicating high accuracy in distinguishing children with long COVID from controls.

These findings support the hypothesis that paediatric long COVID is associated with a combination of identifiable clinical and biological risk factors rather than representing a random post-infectious outcome.

## 4. Discussion

In this retrospective case–control study, we developed an exploratory predictive model for pediatric long COVID based on readily available demographic, clinical, and laboratory variables. The model demonstrated excellent discriminative performance, with an area under the curve (AUC) of 0.94, indicating a high capacity to distinguish children who developed persistent long COVID symptoms from those who recovered completely after acute SARS-CoV-2 infection. These findings reinforce the emerging concept that pediatric long COVID is not a random post-infectious phenomenon, but rather the result of a complex interaction between demographic, clinical, and biological factors [14,15,16].

Data regarding the clinical characteristics of hospitalized pediatric patients with infectious diseases in Eastern Europe remain limited. Previous national studies have shown that demographic and biological factors significantly influence disease expression in Romanian pediatric cohorts [17]. Age emerged as an important contributor to long COVID risk in our cohort, with children in the long COVID group being significantly older than controls. This observation is consistent with recent large pediatric cohort studies reporting an increased prevalence and persistence of long COVID symptoms with advancing age, potentially reflecting age-dependent differences in immune response, symptom perception, and communication abilities [16]. Although sex was not a significant predictor in our population, previous studies have reported inconsistent associations, suggesting that sex-related differences may be cohort-specific or modulated by age and symptom reporting [18]. In Perestiuk et al.’s research, female sex was more likely to be associated with post-COVID persistent symptoms [14]. Urban residence was associated with a higher probability of long COVID in our predictive model. While limited pediatric data are available regarding the influence of living environment, this finding may reflect differences in exposure intensity, environmental stressors, healthcare access, or follow-up practices. Similar associations have been described in adult long COVID populations, where urban living has been linked to increased symptom persistence, possibly mediated by socioeconomic and environmental factors [19]. There is literature regarding the implications of vaccinations in terms of developing long COVID in children, although it might seem reasonable to consider that, since vaccination reduced COVID-19 severity in children [20,21], LCS should be less experienced by those being vaccinated than among children who were not. Our study did not identify any difference regarding vaccination status between long COVID patients and controls. Studies from the United States indicate that disease severity during the acute phase, underweight status, and pre-existing comorbidities, including cancer and cirrhosis, may increase the risk of long COVID [22]. Importantly, acute COVID-19 severity was not directly associated with the development of long COVID in our cohort, as children with asymptomatic or mild initial infections were more likely to develop persistent symptoms. Data from a large Romanian retrospective study conducted during the Omicron wave, including 613 infants hospitalized with symptomatic SARS-CoV-2 infection, showed predominantly respiratory involvement (76.0%), but also frequent digestive manifestations (63.3%). Notably, digestive symptoms were associated with a 1.9-fold increased risk of hepatic cytolysis, while infants aged 1–3 months had a 1.5-fold higher risk of elevated alanine aminotransferase levels and longer hospitalization [23].

This finding aligns with recent evidence indicating that long COVID can occur independently of acute disease severity, challenging earlier assumptions that persistent sequelae are confined to severe initial infections [18]. Instead, symptom burden and biological responses during the acute phase may play a more relevant role than traditional severity classifications. Although the association did not reach statistical significance in our cohort, the higher prevalence of comorbidities among children with long COVID is consistent with previous large cohort and meta-analytic data, indicating that baseline health status may increase susceptibility to persistent long COVID manifestations. López-León et al. identified pre-existing medical conditions, including asthma and allergic diseases, among the most frequently reported risk factors for prolonged symptoms in pediatric populations [24], while Borch et al., in a nationwide cohort study, observed longer symptom duration among children with underlying conditions [25].

Biological parameters contributed substantially to the predictive performance of the model. Children with long COVID exhibited significantly lower lymphocyte and monocyte counts, alongside higher D-dimer, ferritin, and creatinine levels. These findings support the hypothesis that persistent immune dysregulation, low-grade inflammation, and subtle metabolic or endothelial alterations may underlie the pathophysiology of pediatric long COVID [15,26]. Elevated ferritin and D-dimer levels, even within age-adjusted reference ranges, may indicate persistent inflammatory activation or procoagulant tendencies, consistent with observations in both pediatric and adult long COVID cohorts [19,27]. Moreover, preliminary cytokine profiling data suggest that pediatric long COVID may be associated with a pro-inflammatory signature, including altered levels of CCL23 and related immune mediators [28].

The imaging patterns observed in our cohort—more frequent use of lung ultrasound and chest radiography in children with long COVID, but predominantly normal findings—are consistent with emerging pediatric data. A prospective case–control study found that lung ultrasound scores did not differ significantly between children with persistent symptoms and those who fully recovered after SARS-CoV-2 infection, with the majority showing normal or minimal artefacts on LUS [29]. Another pediatric investigation similarly reported an absence of pathological findings on lung ultrasound in subjects with long-term symptoms of long COVID. These results align with earlier pediatric radiology research indicating that structural pulmonary abnormalities (e.g., ground-glass opacities on CT) are more prominent during the acute phase but are not consistently observed on follow-up imaging in children with mild or moderate initial disease [30]. In contrast, abdominal ultrasound was significantly more frequently performed in the control group, likely reflecting the high burden of acute or non-COVID-related gastrointestinal conditions in routine pediatric hospital practice. Gastrointestinal disorders remain a common cause of clinical evaluation in Romanian pediatric settings, as previously illustrated by single-center data on the management of pediatric Helicobacter pylori infection [31]. This contextual factor may explain the higher rate of abdominal imaging among controls and underscores the importance of interpreting imaging utilization within the broader clinical spectrum of hospital presentations.

Consequently, while imaging is often pursued when symptom persistence raises clinical concern, its yield for identifying persistent structural lung pathology in long COVID appears limited in pediatric populations.

Regarding risk factors for long COVID syndrome (LCS), multiple studies have consistently identified older age, female sex, pre-existing medical conditions, and allergic or atopic diseases as important determinants of symptom persistence following acute SARS-CoV-2 infection. The discriminative performance of our multivariable model (AUC = 0.94) compares favourably with previously published pediatric predictive approaches. Perestiuk et al. developed a logistic regression model incorporating demographic, clinical, and laboratory variables, reporting good but more moderate discriminative capacity, supporting the feasibility of structured risk stratification in pediatric long COVID [14]. In contrast, large cohort initiatives such as the RECOVER Consortium have identified clusters of risk factors and symptom patterns but have not yet translated these findings into simplified, clinically applicable risk scores with defined probability thresholds [18]. The high AUC observed in our study may reflect the integration of objective biological parameters alongside clinical variables, reinforcing the added value of laboratory markers in improving model discrimination. In a comprehensive systematic review and meta-analysis including both pediatric and adolescent populations, López-León et al. reported that older age and female sex were among the most frequently associated risk factors for long COVID, alongside a higher prevalence of comorbid conditions such as asthma and other allergic diseases, suggesting a potential role of immune predisposition in post-viral symptom persistence [24]. Similarly, in a large nationwide cohort study from Denmark, Borch et al. evaluated SARS-CoV-2-positive children and reported that persistent symptoms were more common in older children and adolescents, with fatigue, headache, and cognitive difficulties being the most frequently reported manifestations. The authors also observed longer symptom duration among children with pre-existing conditions, further supporting the contribution of baseline health status to long COVID risk [25]. In addition, a systematic review by Notarte et al. examined the impact of COVID-19 vaccination on the development and course of long COVID and reported a reduced risk of persistent symptoms among vaccinated individuals compared with unvaccinated populations [32]. The development of predictive tools for pediatric long COVID should be integrated into structured longitudinal care pathways, similar to established transition models developed for other chronic pediatric conditions [33] demonstrated to improve adherence and reduce loss to follow-up. Although most included studies involved adults, the findings suggest that vaccination may mitigate long COVID risk by reducing viral load, systemic inflammation, and immune dysregulation during acute infection; mechanisms that are also relevant to pediatric populations [32]. Collectively, these data indicate that long COVID risk is influenced by a combination of host-related factors, including age, sex, baseline immune and allergic status, and pre-existing comorbidities. This multifactorial risk profile supports the rationale for predictive modelling approaches that integrate demographic, clinical, and biological variables to identify children at increased risk for persistent post-COVID symptoms. Nevertheless, the absence of external validation may have contributed to optimistic performance estimates, and replication in independent cohorts is required to confirm generalizability.

The present research did not evaluate the impact of specific pharmacological treatments used for COVID treatment, such as antiviral or antibacterial agents, anticoagulants, or corticosteroids, on the development of long COVID syndrome in children, due to limited sample size and driven by relative low incidence of using these therapeutic interventions.

A major strength of this study lies in the integration of objective laboratory markers into the predictive model. While most pediatric long COVID studies rely primarily on symptom-based definitions, our results highlight the added value of biological parameters in risk stratification. Similar multivariable approaches have been proposed in the recent literature, emphasizing their potential utility in guiding early follow-up, multidisciplinary assessment, and personalized care strategies [14].

Several limitations should be acknowledged. The retrospective, single-center design may limit generalizability, and external validation in independent pediatric cohorts is required. The retrospective design of the study and the relatively limited number of events compared to the number of analyzed variables may have increased the risk of model overfitting. Therefore, the identified associations and the proposed scoring approach should be considered exploratory and hypothesis-generating, requiring prospective validation in larger pediatric cohorts before clinical application. Additionally, the absence of advanced immunological profiling or longitudinal biomarker assessment restricts mechanistic interpretation. Nonetheless, the excellent discriminative performance of the model supports its potential clinical relevance and warrants further prospective evaluation. Further external validation through large multicenter prospective pediatric cohorts is necessary to assess the reproducibility, calibration, and clinical utility of the proposed exploratory model across different populations and healthcare settings before routine clinical implementation. Overall, these findings contribute to the growing body of evidence supporting pediatric long COVID as a multifactorial condition with identifiable risk factors. Predictive modelling approaches such as the one presented here may facilitate earlier identification of at-risk children and support targeted follow-up strategies, with potential benefits for long-term outcomes and healthcare resource optimization.

## 5. Conclusions

This study demonstrates that a multivariable predictive model incorporating clinical and laboratory parameters can accurately identify children at risk of developing long COVID. The discriminative performance of the model underscores the relevance of combining demographic factors, comorbidities, symptom profiles, and biological markers in pediatric long COVID risk stratification. These findings support the implementation of structured long COVID follow-up protocols and highlight the need for prospective validation of predictive tools to inform clinical decision-making in pediatric practice.

## Figures and Tables

**Table 1:** Baseline characteristics of the study groups.

Characteristic	Long COVID (n = 89)	Control Group (n = 88)	*p* Value
Age (years), mean ± SD	1.79 ± 0.90	1.14 ± 0.80	0.001
Sex, n (%)			0.41
– Male	42 (47.2%)	48 (54.5%)	
– Female	47 (52.8%)	40 (45.5%)	
Living environment, n (%)			0.0099
– Urban	77 (86.5%)	61 (69.3%)	
– Rural	12 (13.5%)	27 (30.7%)	
Comorbidities, n (%)			0.071
– Without comorbidities	59 (66.3%)	77 (87.5%)	
– With comorbidities	30 (33.7%)	11 (12.5%)	
COVID-19 vaccination, n (%)			1.00
– Yes	1 (1.1%)	1 (1.1%)	
– No	88 (98.9%)	87 (98.9%)	

**Table 2:** Laboratory parameters in children with long COVID and controls.

Parameter	Long COVID (Mean ± SD)	Control Group (Mean ± SD)	*p* Value
Total leukocytes	9.23 ± 3.30	9.24 ± 3.93	0.98
Neutrophils	3.25 ± 1.63	3.38 ± 1.60	0.58
Lymphocytes	3.85 ± 1.47	4.51 ± 1.88	0.010
Eosinophils	0.37 ± 0.27	0.40 ± 0.35	0.46
Monocytes	0.58 ± 0.19	0.92 ± 0.40	<0.001
Basophils	0.03 ± 0.02	0.10 ± 0.07	<0.001
Platelets	305.38 ± 56.92	302.38 ± 114.88	0.83
Hemoglobin	12.59 ± 0.70	11.10 ± 1.18	<0.001
CRP	15.50 ± 21.07	17.96 ± 25.08	0.48
ESR	1.24 ± 11.66	0.00 ± 0.00	0.32
Fibrinogen	327.78 ± 91.57	345.84 ± 96.66	0.20
D-dimers	51.07 ± 123.61	10.39 ± 46.24	0.004
Serum iron	34.19 ± 47.84	14.65 ± 23.12	0.001
Ferritin	49.62 ± 68.76	19.25 ± 32.83	<0.001
ALT	31.02 ± 47.12	25.49 ± 24.15	0.33
AST	54.65 ± 51.74	46.02 ± 31.25	0.18
Urea	22.59 ± 5.26	20.93 ± 5.91	0.049
Creatinine	0.42 ± 0.23	0.29 ± 0.08	0.001

**Table 3:** Variables and coefficients included in the COVID-19 risk score model.

Variable Category	Variable Definition	Regression Coefficient (β)	Contribution to Score (β × Value)
Intercept	–	1.349	+1.349
Age	Years	0.339	+0.339 × Age
Living environment	Rural (0)/Urban (1)	0.641	+0.641 × Living environment
Comorbidities	Absent (0)/Present (1)	0.683	+0.683 × Comorbidities
COVID-19 severity	Asymptomatic (0); Mild (1); Moderate (2); Severe (3); Critical cases (4)	−1.307	−1.307 × Severity
Lymphocytes (Ly)	Absolute lymphocyte count	1.211	+1.211 × Ly
Monocytes (Mo)	Absolute monocyte count	−12.266	−12.266 × Mo
D-dimer	D-dimer level	0.013	+0.013 × D-dimer
Ferritin	Serum ferritin level	0.027	+0.027 × Ferritin
Creatinine	Serum creatinine level	6.974	+6.974 × Creatinine

## Data Availability

The datasets generated and/or analyzed during the current study are not publicly available due to patient confidentiality and ethical restrictions involving pediatric patient data.
